# Effects of microplastic exposure on the body condition and behaviour of planktivorous reef fish (*Acanthochromis polyacanthus*)

**DOI:** 10.1371/journal.pone.0193308

**Published:** 2018-03-01

**Authors:** Kay Critchell, Mia O. Hoogenboom

**Affiliations:** 1 College of Science and Engineering, James Cook University, Townsville, Australia; 2 Centre for Tropical Water and Aquatic Ecosystem Research, James Cook University, Townsville, Australia; 3 ARC Centre of Excellence for Coral Reef Studies, James Cook University, Townsville, Australia; Department of Agriculture and Water Resources, AUSTRALIA

## Abstract

The effect of a pollutant on the base of the food web can have knock-on effects for trophic structure and ecosystem functioning. In this study we assess the effect of microplastic exposure on juveniles of a planktivorous fish (*Acanthochromis polyacanthus*), a species that is widespread and abundant on Indo-Pacific coral reefs. Under five different plastic concentration treatments, with plastics the same size as the natural food particles (mean 2mm diameter), there was no significant effect of plastic exposure on fish growth, body condition or behaviour. The amount of plastics found in the gastro-intestinal (GI) tract was low, with a range of one to eight particles remaining in the gut of individual fish at the end of a 6-week plastic-exposure period, suggesting that these fish are able to detect and avoid ingesting microplastics in this size range. However, in a second experiment the number of plastics in the GI tract vastly increased when plastic particle size was reduced to approximately one quarter the size of the food particles, with a maximum of 2102 small (< 300μm diameter) particles present in the gut of individual fish after a 1-week plastic exposure period. Under conditions where food was replaced by plastic, there was a negative effect on the growth and body condition of the fish. These results suggest plastics could become more of a problem as they break up into smaller size classes, and that environmental changes that lead to a decrease in plankton concentrations combined with microplastic presence is likely have a greater influence on fish populations than microplastic presence alone.

## Introduction

Plastic pollution has been reported in every ocean and sea on Earth [1], and is widely recognised as a global threat to marine life, and to the economies of coastal nations [2, 3]. Plastic pollution can degrade coastal benthic habitats through smothering, when plastic sheets form a layer over the benthos and, also, through changes in sediment permeability due to buried plastics [4]. Moreover, plastic pollution causes harm to wildlife through entanglement and ingestion [2]. Although plastics do not readily biodegrade, they do break up into smaller pieces when exposed to ultraviolet light and physical abrasion [5]. As plastic particles become smaller (i.e. microplastics, less than 5 mm in diameter) they become available to be inadvertently consumed by a wide range of marine organisms. Ingestion of plastics has been reported in many species of marine fauna, most notably seabirds [6, 7] and sea turtles [8], but also fish [9–11], corals [12], and other invertebrates [13–15]. Consumption of microplastic by organisms at the base of food webs, such as mussels [16] and plankton [17], has raised concerns about the potential for transfer of plastic-associated toxins throughout marine food webs [3].

Ingested plastics can cause harm though physical damage to the gastrointestinal (GI) tract. For instance, plastic ingestion can cause abrasions and lesions, or physical disruption of the GI tract, as plastics compact in the gut (e.g. [18]). Plastic ingestion can also be detrimental to the health of various organisms because indigestible particles fill the stomach and reduce the feeling of hunger which leads to starvation (e.g. [19, 20]). Conversely, microplastics (defined as plastic fragments < 5 mm [5]) have been found in the stomach or GI tract of marine animals without causing obvious harm. For example, 12.2% of harbour seals assessed in the Netherlands contained microplastics, with no clear effect of the plastic consumption on the animals [21]. However, many studies to date have simply reported the presence of plastics in the GI tract without assessing the effects on the fitness of the organism [17, 22]. Overall, while there is growing evidence that many different taxa consume microplastic particles, the potential health effects of such ingestion are not well known.

In addition to the physical effects of plastics on animal digestion, many plastics contain chemicals, such as flame retardants, which are added during the production process to give the plastics certain properties [2, 23, 24]. Plastic additives can be transferred into the tissues of animals that have consumed plastics [25, 26], with potential effects on the physiology and health of the organism. Laboratory experiments have been used to assess the effect of plastic consumption on the development and body condition of oganisms [27]. For example, lugworms in an experimental trial were found to lose weight over a 28 day exposure to microplastics with polychlorinated biphenyls (PCBs) [28], but it remains unclear whether it is the physical presence of microplastics, or the toxic effects of PCBs, that cause this effect. PCBs can alter the regulation of key hormones including estrogen, testosterone and thyroxine [29, 30]. Changes in hormone concentrations can have complex effects on animal behaviour. Independent of toxicological effects, changes in behaviour can also be expected in response to starvation which may result from plastic ingestion. For instance, a hungry individual may become more aggressive or listless; it may also increase territoriality [31, 32, 33]. de Sá et al. [34] recently showed that the ingestion of plastics effects the predatory behaviour of the common goby, however the mechanism underlying this change in behaviour remains unknown. Despite the important role of animal behaviour in determining performance in the natural environment, the effects of microplastic exposure and/or ingestion on animal behaviour remain largely unknown.

Although plastic consumption by marine vertebrates is arguably best documented in seabirds [35], many studies have also demonstrated that teleost fish consume microplastics in the natural environment [9, 11, 36–38]. For seabirds, some species are reported to have up to 80% of individuals containing plastics [39], and the mortality from starvation, which occurs when birds consume plastics instead of their normal food, is obvious when seabird carcasses are observed on beaches [40]. For fish, field studies have revealed that up to 30% of individuals have plastics in their GI tracts [37, 41]. However, some species of fish generally retain low numbers of plastic particles per fish (two to four) [37, 41]. Moreover, plastics consumed by fish tend to be small in size, with 22 of 121 fish gut-content samples from the Mediterranean containing plastics, of which 70% were < 5 mm [41]. Nevertheless, it is currently unknown whether ingestion of small quantities of microplastics is detrimental to the health of these fish. To date, field studies of fish plastic ingestion have primarily focused on pelagic and commercially important species, including mackerel [10, 38, 41, 42]. These fish prey on smaller species of fish and it is unclear whether these piscivorous fishes consume microplastics directly from the water column, or whether they incidentally ingest plastics by consuming prey that had eaten plastics themselves. Understanding the level of fish stock contamination by plastics requires an understanding of these trophic links.

There is high variation in fish diets, both within and among species. This difference stems from feeding strategy or natural prey size differences [43, 44]. Some species have a highly selective diet [44, 45], suggesting that such species might only rarely eat plastics in their natural environments. For example, Carpenter et al. [46] showed that only white spheres were found in the gut of 8 diffferent fish species, indicating selective feeding on white spheres over other types of plastics. Fish also display ontogenetic changes in diet as they grow larger, with smaller fish generally eating smaller prey [47]. For instance, some damselfish species increase their reliance on consumption of benthic algae as they mature [48], meaning that juvenile fish that consume plankton may be more at risk of harm from microplastic consumption than adults. In addition to ontogenetic shifts in diet, fish show individual variation in their feeding behaviour [49], and might differ in their propensity to consume plastics. Feeding behaviour can also be determined by the odour of biofilm on plastic debris [50]. Understanding effects of plastic ingestion on fish populations therefore requires quantification of among-individual variation in the propensity to ingest plastic, and in the effects of plastic ingestion.

The effects of plastic exposure on fish growth and behaviour are likely to be concentration-dependent. It is our hypothesis that if plastic consumption depends on plastic availability in seawater, greater plastic ingestion, and greater potential impacts of plastic feeding, should be observed at higher plastic concentrations. Similarly, if plastic replaces plankton in seawater this is likely to alter fish growth and behaviour through starvation effects. In this study, we aimed to quantify whether and how plastic ingestion by fish depends upon plastic concentration, and to assess the effects of plastic consumption on the growth and body condition of juvenile planktivorous reef fish. We also aimed to determine whether plastic consumption and/or plastic presence in seawater affected fish behaviour, to test the hypothesis that an increase in the concentration of a poor food resource (plastics) lead to an increase in aggressive behaviour due to more frequent failed foraging efforts. Our final aim was to assess the likelihood of plastic consumption with different size classes of plastics for different size classes of fishes. It was our hypothesis that reduced plastics size would increase the consumption of plastics as the ability to distinguish between food and non-food particles was reduced.

## Methods

### Ethics statement

This study was carried out in strict accordance with ethics protocol laid out by the *Animal Ethics Committee of James Cook University*, who approved the protocol of these experiments (Permit number: A2112), and priority was given to animal care at all stages of this study.

### Overview of approach

An experimental approach was used to isolate the effects of plastic consumption for a common planktivorous reef fish under otherwise controlled conditions. We chose juvenile *Acanthochromis polyacanthus* as a representative species. *A*. *polyacanthus* is a geographically widespread planktivorous reef fish, common throughout the Great Barrier Reef (GBR), and like most planktivores, has relatively low feeding selectivity [48], making them a good candidate for feeding trials. Also, *A*. *polyacanthus* is a commonly used experimental fish species as they are easy to rear and care for in a laboratory environment.

**Fig 1:**
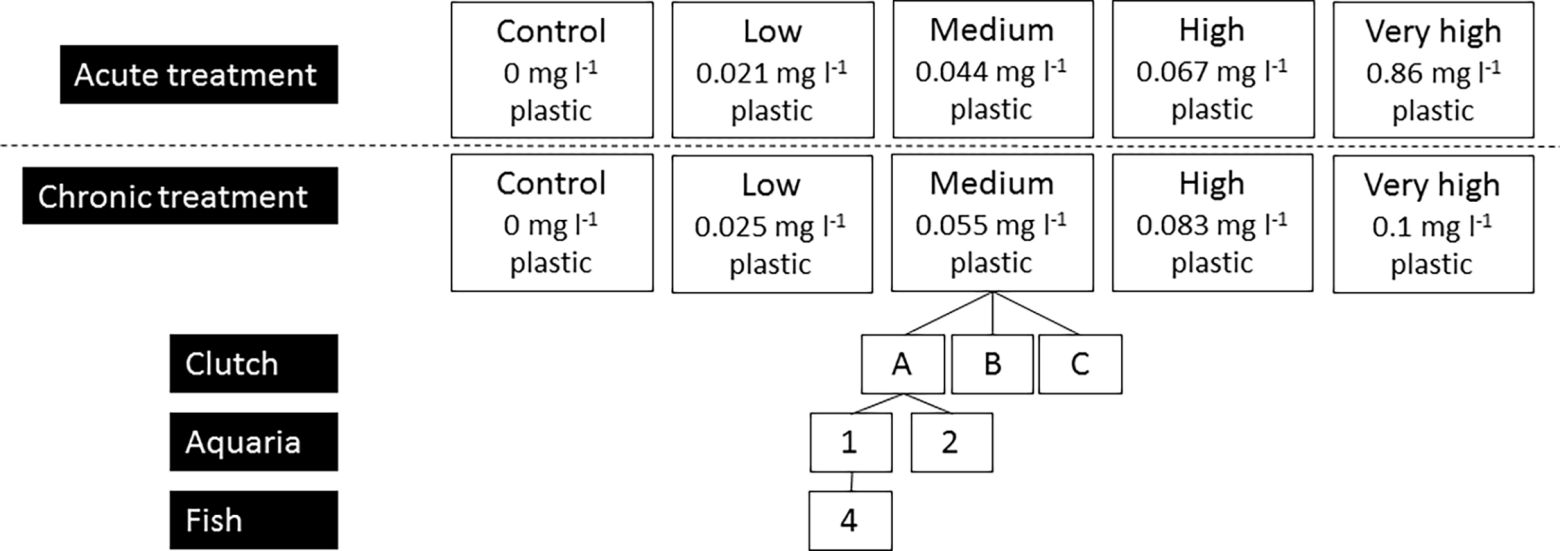
Experimental design for acute and chronic plastic exposure experiment. Concentrations shown are the mean concentrations for each treatment, as treatment dosage was dependent on tank biomass.

We conducted an aquarium-based experiment to determine whether plastic ingestion affected the growth and body condition, and behaviour of juvenile *A*. *polyacanthus*, and whether any effects were concentration dependent (Fig 1). This experiment consisted of two phases. First, we assessed whether replacement of food by plastic was detrimental to fish growth (referred to hereafter as ‘acute exposure’). Second, we assessed the influence of exposure to plastics in addition to a normal level of food which is adequate for fish growth and development (referred to hereafter as ‘chronic exposure’). To minimise the number of animals exposed to plastics (consistent with animal ethics guidelines), these phases were implemented sequentially, with one week of acute exposure followed by 6 weeks of chronic exposure. Growth rates (length and mass) were measured weekly. A separate aquarium experiment was conducted to assess whether ingestion of plastic by fish depended on plastic particle size. In that experiment we exposed fish of two size classes to three size classes of plastic particles for one week, after which their gut content was analysed to assess the amount of plastic retained.

Experiments were conducted in a large recirculated marine aquarium system at the Marine and Aquaculture Research Facility (MARF) at James Cook University. Water in aquaria was maintained at a temperature of 27.5°C (±1°C), neutral pH, salinity of 36 ppt, and with nitrates (NO_3_) within the range 28.5 and 32 mg l^-1^. Juvenile *A*. *polyacanthus* (N = 112) from three sets of parent stock (hereafter, ‘clutches’, labelled A-C) that had been raised in captivity at MARF, were reared between February and April 2015. Juvenile fish were raised at low stock densities and were fed a commercial, high nutrition food twice per day *ad libitum* until the cohort average length (fork length) was approximately 3.5 cm. The fish from each clutch were randomly split into one of five treatments with different microplastic concentrations: control (0 mg l^-1^ plastic), low (average 0.025 mg l^-1^ plastic), medium (average 0.055 mg l^-1^ plastic), high (average 0.083 mg l^-1^ plastic) and very high (average 0.1 mg l^-1^ plastic). There were two replicate aquaria per clutch, per treatment for a total of six replicate aquaria per treatment (n = 30 aquaria). During the experiment the fish were fed the same type of commercial food pellets as the growth phase, and the food amount was adjusted according to fish biomass in each aquarium (g of food = fish biomass * 0.0125) because the amount of food required for tissue maintenance and growth by fish depends on their biomass [51]. The amount of plastic added to tanks was also adjusted according to fish biomass in the aquaria, so that there was a constant provision of plastic per unit fish biomass within each treatment (g of plastic = g of food/100 * treatment; 0, 20, 40, 60, 80). Differences in fish biomass among clutches, and over time, meant that the amount of plastics provided to tanks overlapped slightly between treatments (see Table A in S1 File for weekly food and plastic concentrations per treatment). The objective of this experiment was to assess the dose response to plastic exposure. The wide range of concentrations used here are a common approach in eco-toxicological assessments [52], and are required to quantify response thresholds where they exist. Fish were fed twice a day for the duration of the experiments and were left to feed for at least four hours after exposure. After four hours the aquaria were cleaned to remove as much of the uneaten plastics as possible, this was to ensure each feed was the precise concentrations required for the treatment. Each replicate aquarium housed four fish, except for clutch B where only 32 individuals were available, and therefore, aquaria housed three or four individuals (Fig 1). Total fish replicates per treatment therefore varied slightly (N_control_ = 23, N_low_ = 22, N_medium_ = 22, N_high_ = 23, and N_veryHigh_ = 22). All aquaria contained short sections of PVC pipe at the base of the aquarium as shelters for the fish and mesh over the outflow (and aquaria tops) to prevent fish loss. Individual fish were tagged using elastomer tags to enable measurement of growth of each individual fish over the experimental period.

### Preparation of microplastics

Polyethylene terephthalate (PET) was used for the experiment as it is one of the most common plastics types found in the environment [53]. For consistency with the food supplied to the fish during rearing, and the size of their natural prey, PET microplastics with a particle size approximately the same as the commercial food pellets were created by cryomilling post-consumer recycled plastic pellets, donated from Visy Plastics (Smithfield, NSW, Australia). The cryomilling process created a range of particle sizes, the desired size classes were obtained by sieving the raw cryomilled particles. The commercial food pellets were approximately 2 mm in diameter and the plastics ranged from 1 to 2 mm. The plastic particles were divided into mesh bags and placed in indoor sump aquaria of a mature salt water aquarium system for at least two weeks prior to the start of the experiment. This was to encourage the growth of a microbial biofilm on the surface of the milled microplastics to better mimic microplastics in nature [50]. PET has a specific gravity of 1.38 and is, therefore, negatively buoyant in seawater. However, the small milled particles have a high surface area to volume ratio and remained at the water surface for ~2 minutes after being dropped into the water, behaving similarly to the commercial food pellets.

### Acute exposure experiment–food replaced by plastic

During the first week of the exposure experiment, we assessed the impact that replacement of natural food products with plastic particles might have on fish. This ‘acute’ exposure was designed to indicate how fish growth might be affected in marine habitats where natural plankton concentrations are declining (evidence in [54]) and microplastic concentrations are increasing [55]. During this week, the fish (N = 112) received a total ‘food’ allowance between 0.022 g to 0.065 g (0.055–0.16 mg l^-1^) per feed, depending on aquaria fish biomass, which included different proportions of food and PET (see Table A in S1 File).

### Chronic exposure experiment–plastic dose added to normal food

After the ‘acute’ exposure phase, fish were exposed to plastic concentrations which consisted of their normal food (biomass adjusted) plus either zero (control group), low (20%), medium (40%), high (60%) or very high (80%) percentage of their diet added as plastic (see Table A in S1 File). No tank received more than 0.065g (0.16 mg l^-1^) food per feeding bout, as excess food in the system can reduce water quality.

### Ontogenetic changes in sizes of microplastics ingested

A second group of fish (n = 69) were sourced from stock *A*. *polyacanthus* at MARF and were divided into two size classes, based on fork length, with small fish 30 to 35 mm and large fish 35 to 45 mm. Fish from these two treatment groups were randomly allocated among three different plastic particle size treatments (small, 125–300 μm [approx. 140000 per g]; medium, 300–1000 μm [approx. 5000 per g]; and large, 1000–5000 μm [approx. 60 per g]), with 3 replicate aquaria per treatment with three to four fish per aquarium. The fish were fed twice daily with a diet of commercial pellets (amount calculated based on fish biomass in each aquaria as above) with additional plastic 80% of the food mass (0.05 to 0.13 mg l^-1^ per feed, see Table B in S1 File). We acknowledge that using weight of particles resulted in a different number of particles being supplied to each tank depending on particle size. The aim of using high concentrations of particles was an attempt to make consumption rate limited by particle processing time, rather than the number of particles available (a type II functional response) [56]. After one week exposure, the fish were euthanized and dissected to assess the amount of plastic retained in the whole length of the GI tract of each animal.

### Measured response variables

#### Plastic retention, growth and body condition

At the conclusion of the plastic exposure experiments, the fish were euthanized according to standard animal ethics protocols, by MS-222 overdose. The gut content of the fish was collected to determine the amount of plastics the fish retained. This plastic ingestion quantifies the amount of plastic ingested and retained in the gut over both the acute and chronic phases of the exposure experiment. The same procedure was followed at the end of the seven day duration particle size experiment.

We collected weekly length and weight data to assess growth through the acute and chronic exposure phases. Each fish was photographed using a camera fixed a set distance from a gridded background, to accurately measure length and width using ‘Image J’ software. The wet weight of each fish was measured by adding the fish to a beaker of aquarium water on pre-zeroed scales (Kern PBC 3-place analytical balance, Kern and Sohn KmbH, Germany). Individual growth rates were calculated, as change in mass and change in length, weekly and over the duration of the whole experiment. Length weight ratios were calculated for each fish at the beginning and end of both acute exposure and chronic exposure phases. The change in this ratio gives an indication of the change to the body condition of each fish over each phase of the experiment. During the post exposure dissections, the liver of each fish was extracted and weighed (ME235P, 5-place Sartorius analytical balance, Germany) to calculate the liver weight to body weight ratio (hepatosomatic index; Eq 1), used to assess the body condition of each fish [57].

$$Hepatosomatic\;index\;(HSI)=\left(\frac{LW}{BW}\right)\times 100$$ Eq 1

Where LW is liver weight and BW is the total body weight of the fish before dissection. The HSI reflects the energy reserves in the liver and, as liver function is critical for overall health, is a reliable proxy for condition [58].

#### Behaviour

Effects of microplastic exposure on fish behaviour were assessed using two sets of video observations at the end of the chronic exposure phase. These video observations monitored: 1) fish swimming activity and aggression between feeding times (20 minute videos, taken in between feeding times between the hours of 10 am and 4 pm); and 2) foraging behaviour and aggression at feeding times, for two minutes after food was introduced to each aquarium. To ensure the fish were behaving “normally” after the disruption of camera placement, the camera was set up and set to record 10 minutes before the introduction of food. We used a GoPro (Hero 3) camera that was affixed to a stand that kept the camera at a set distance from the bottom of the aquaria.

Fish swimming activity was measured between feeding times, using the ‘grid crossings’ method [59]. Briefly, a 2 x 2 cm grid (corresponding to approximately one body length of *A*. *polyacanthus* in our videos) was placed over the computer screen during video playback, and the number of times each individual fish crossed a grid line was counted during a period of 2 minutes. The fish was considered to have crossed a gridline if the nose and both eyes crossed the line. Data are reported as line crossings per second. For this metric we used the longer inter-feeding videos with the 2 min sample at least 10 min after the start of the video to ensure the behaviour had returned to “normal” after camera set up.

Aggressive behaviour between individual fish was assessed from the videos taken during and after feeding, and was measured as active interactions between individuals where a dominant fish caused a subordinate to move position within the aquaria. The total number of aggressive interactions in the focal aquaria were counted during the remainder of the video, which ranged from 5–10 minutes (depending on video length) after the food was introduced. We used the maximum video time available to gain the maximum data, the count was then made relative to time in the aggression index described below (Eq 2). The aggressive interactions observed in each aquarium were categorised into short and long interactions. The short interactions (hereafter called ‘swoops’) were counted as lunges or movements of a dominant fish that caused a subordinate fish to move away, whereas long interactions (hereafter called ‘chases’) were counted as interactions where the dominant fish chased a subordinate fish for approximately ¼ the aquaria length (assessed visually; not measured for every interaction). An aggression index (AI) was calculated to account for the different energy costs of swoops compared with chases, due to the different levels of swimming activity and different duration of activity, and was normalised by the duration of the observation period as:

$$AI=\frac{\left(S+2C\right)}{t}$$ Eq 2

Where S is the observed number of swoops, C is the observed number of chases, and t is the duration of the observation period (decimal minutes). This creates a measure of aggression experienced by the fish per aquaria.

### Data analysis

Analysis of the length-weight relationship of the experimental fish showed that three different clutches started with different sizes, and different weights per unit body length (See S2 File, ANOVA, F = 747.9, df = 5, 106, p-value < 0.001). Consequently, ‘clutch’ was retained as a factor in subsequent analyses.

To assess differences in plastic ingestion between treatments from the chronic exposure experiment we used a generalized linear mixed effect model, fit by Laplace Approximation maximum likelihood in the lme4 package in R [60], with the Kenward-Roger approximation to obtain p-values. To understand if the number of plastics retained during the chronic exposure varied between treatments, we fit a linear mixed effect model to the plastic consumption data for the subset of fish that ate plastics, again using the nlme package. Due to the small number of fish that consumed plastics, there was insufficient statistical power to analyses these data among individual treatments and, therefore, the treatments were pooled into high (60% and 80%) and low (20% and 40%) treatments for this particular analysis. Consumption levels by fish of different sizes, for each plastics sizes were assessed using the Fisher’s exact test using a multi-level contingency table analysis.

Generalized linear mixed effect models were fitted to growth, body condition, hepatosomatic index, and line crossing data to assess effect of treatment and clutch on fish health, with aquaria included as a random effect. It was important to include aquaria as a random effect to remove the artificial inflation of statistical replicates, while maintaining the individual fish data. These analyses were conducted using the nlme package for R. Linear models using the nlme package were also fit to the length and weight data to quantify the relationships between and within clutches, and the difference in the slope/intercept of the relationship was assessed to understand body condition during the acute phase of the exposure experiment. To test the effect of plastics exposure on the activity of the fish, the line crossings per second were compared using a Levene’s Test for homogeneity of variance. An ANCOVA was conducted to test the relationship between treatment and aggression with the aggression index data.

## Results

### Plastic ingestion

#### Chronic exposure experiment

**Fig 2:**
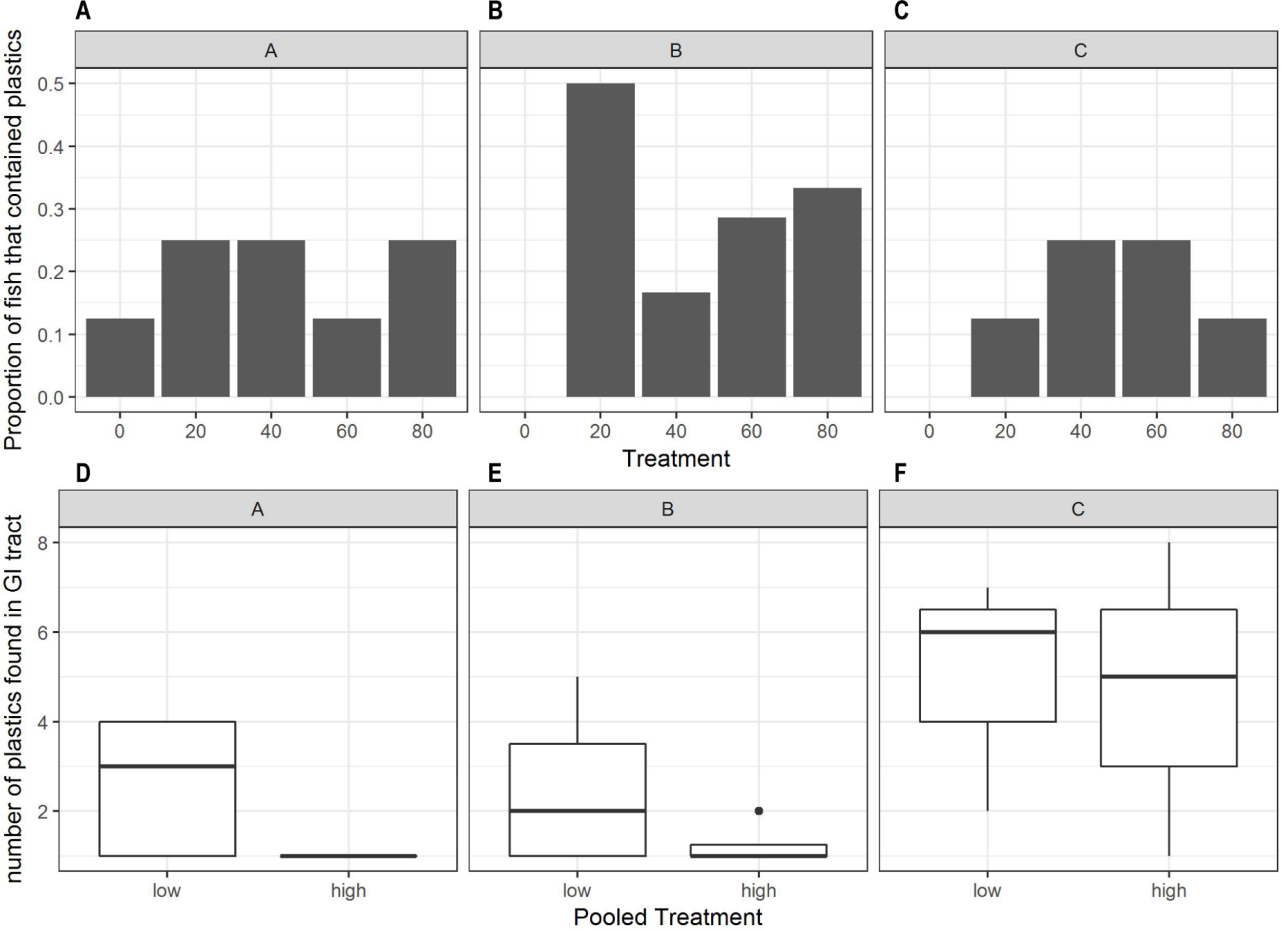
Plastic ingestion by sub-adult *A*. *polyacanthus* exposed to different plastic doses. Top panels (A-C) show proportion of fish per treatment and clutch which ingested plastics, and the lower panels (D-F) show the range of ingestion for fish per pooled treatment (treatments 20, 40 pooled to low, and 60, 80 pooled to high) for each clutch.

At the end of the chronic phase of the exposure experiment, 19.6% of fish had plastic fragments in their GI tracts. For these fish, the number of retained plastic fragments ranged from one to eight (Fig 2) and there was a general trend toward higher ingestion at intermediate plastic exposure concentrations (Fig 2). However, neither the proportion of fish that had retained plastics (Fig 2A–2C), nor the number of plastics retained, were dependent on the concentration of plastic present in aquaria (Fig 2D–2F), and these responses did not vary between clutches (Table 1).

**Table 1:** Summary table of statistical analyses.

Analysis	Factor	Df	Test statistic	p	Fig
Proportion of fish that ingested plastic	Treatment	4,96	*F* = 0.001	0.9	2A-C
	Clutch	2,96	*F* = 0.006	0.9	
	Treatment by Clutch	8,96	*F* = 0.02	0.9	
Number of plastic particles eaten by fish	Intercept	1,11	*F* = 42	<0.001	2D-F
	Treatment (pooled)	1,11	*F* = 1.2	0.29	
	Clutch	2,11	*F* = 4.9	<0.05	
	Treatment by Clutch	2,11	*F* = 0.22	0.80	
Size-dependence of plastic ingestion	Intercept	1,51	*F* = 17	<0.001	3
	Treatment (pooled)	1,12	*F* = 0.70	0.42	
	Clutch	2,12	*F* = 16	<0.001	
	Treatment by Clutch	2,12	*F* = 0.40	0.68	
Growth–acute phase	Intercept	1,82	*F =* 151.4	<0.0001	4A-C
	Treatment	4,15	*F =* 11.1	0.0002	
	Clutch	2,15	*F =* 34.1	<0.0001	
	Treatment by Clutch	8,15	*F = 1*.*66*	0.1888	
Body condition–acute phase	Intercept	1,82	*F =* 33.7	<0.0001	4D-F
	Treatment	4,15	*F =* 6.75	0.0026	
	Clutch	2,15	*F =* 16.01	0.0002	
	Treatment by Clutch	8,15	*F =* 0.74	0.6550	
Growth–chronic phase	Intercept	1,82	*F =* 554.6	<0.0001	5A-C
	Treatment	4,15	*F =* 1.36	0.2938	
	Clutch	2,15	*F =* 36.5	<0.0001	
	Treatment by Clutch	8,15	*F =* 0.58	0.7756	
Body condition–chronic phase	Intercept	1,82	*F =* 786.8	<0.0001	
	Treatment	4,15	*F =* 2.53	0.084	
	Clutch	2,15	*F =* 6.47	0.0094	
	Treatment by Clutch	8,15	*F =* 0.91	0.5317	
Hepatosomatic index	Intercept	1,82	*F =* 2267.8	<0.0001	5D-F
	Treatment	4,15	*F =* 2.66	0.074	
	Clutch	2,15	*F = 4*.*56*	0.0283	
	Treatment by Clutch	8,15	*F = 2*.*17*	0.0926	

#### Ingestion of different particle sizes

**Fig 3:**
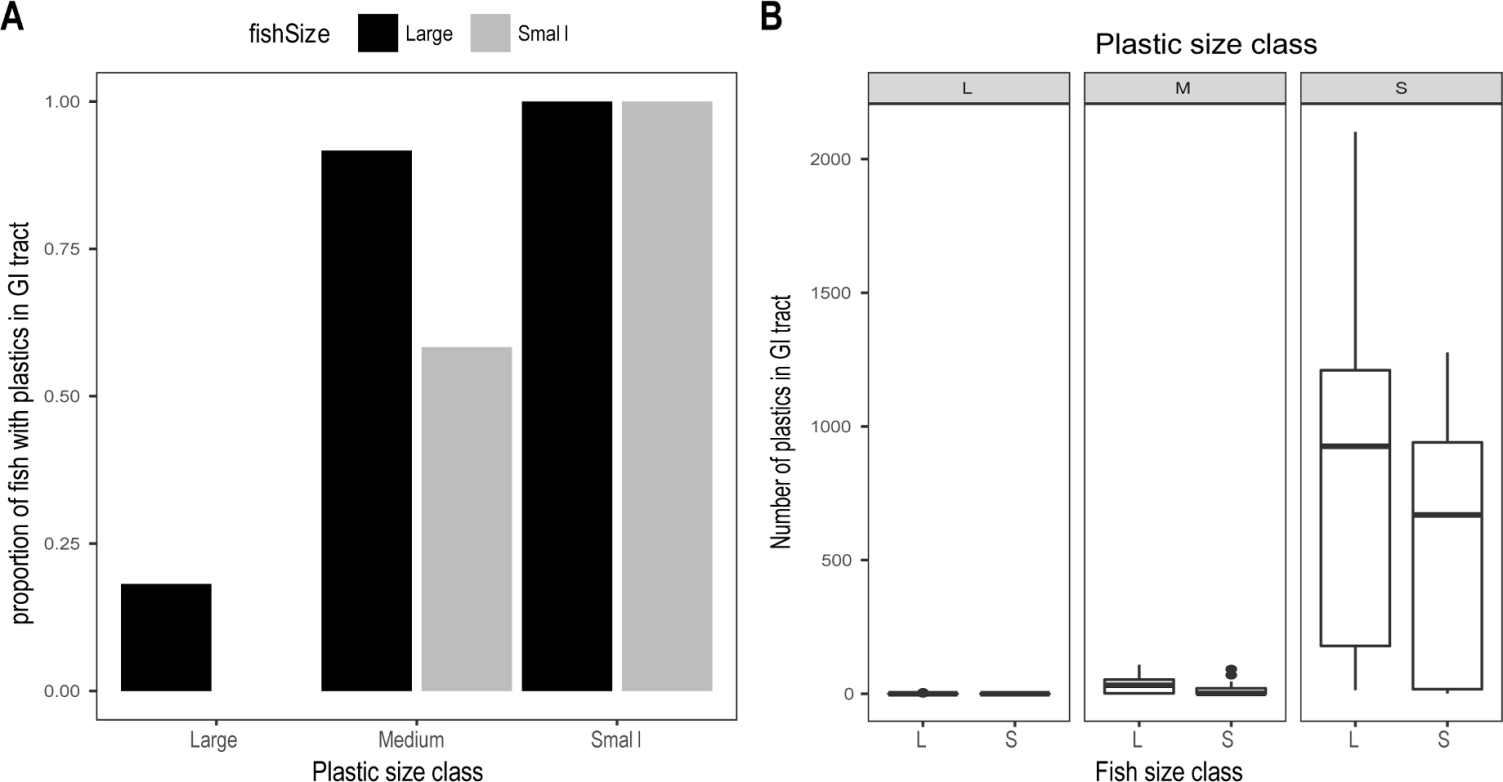
Particle and fish size dependence of plastic ingestion. A) Shows the proportion of fish that were found to have plastics in their digestive tract. B) Shows the range of plastic ingestion for fish per treatment, the mid-line of the boxes represent means with the boxes showing the 25th and 75th percentile and the vertical lines representing the range.

The number of retained plastics was strongly dependent upon plastic particle size. All fish exposed to the smallest plastic fragments were found to have plastics in their GI tract after one week of exposure (Fig 3), and over half of the fish exposed to medium sized plastics were also found to have plastics in the GI tract (Fig 3). The observed proportions of fish of different sizes that had consumed small and large plastics was significantly different than random (Fishers Exact Test, p-value < 0.001). Larger fish appeared to be more likely to ingest plastics of all sizes than smaller fish (Fig 3A). For each particle size treatment there was no statistical difference in amount of plastics consumed between the fish sizes (Fig 3B). However, there was a large difference in the amount of plastic consumed between particle size treatments, with fish ingesting up to a maximum of 2102 small plastic fragments (in a fish classed as large) in comparison to a maximum of 5 in the large plastic treatment (Fig 3). Fish were found to have much higher numbers of plastics in their GI tract when exposed to the small particle treatment (Fig 3B, F = 15.523, df = 12, p-value = 0.0005).

### Growth and body condition

#### Growth over the different exposure regimes

**Fig 4:**
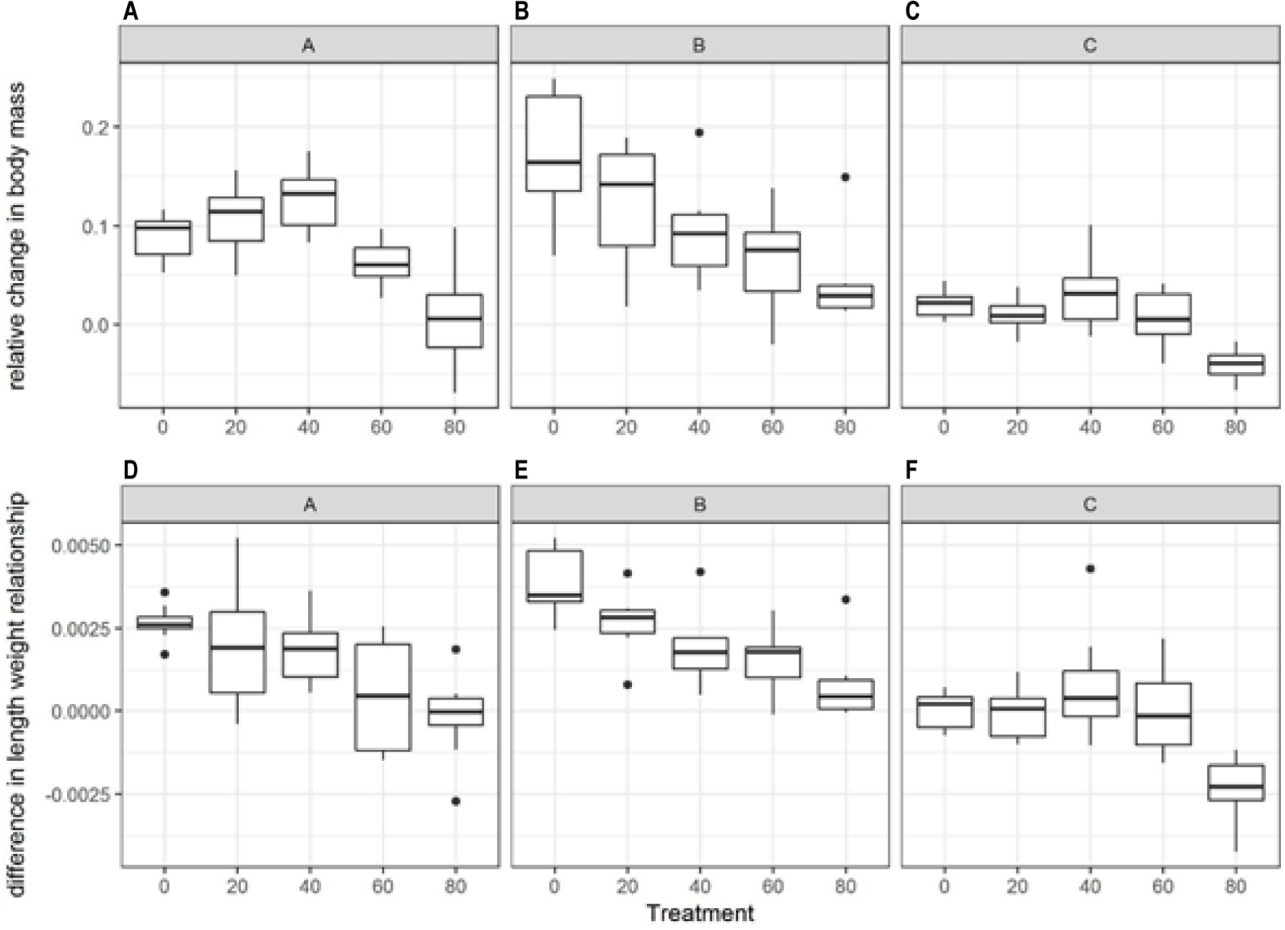
Fish growth and body condition during the acute phase of the plastic exposure experiment. Top panels (A-C) show the change in mass (g) relative to start mass during the acute exposure phase, for each clutch. The lower panels (D-F) show the change in length-weight relationship relative to the start of the acute exposure, for each clutch. The mid-line of the boxes represent means with the boxes showing the 25th and 75th percentile and the vertical lines representing the range.

**Fig 5:**
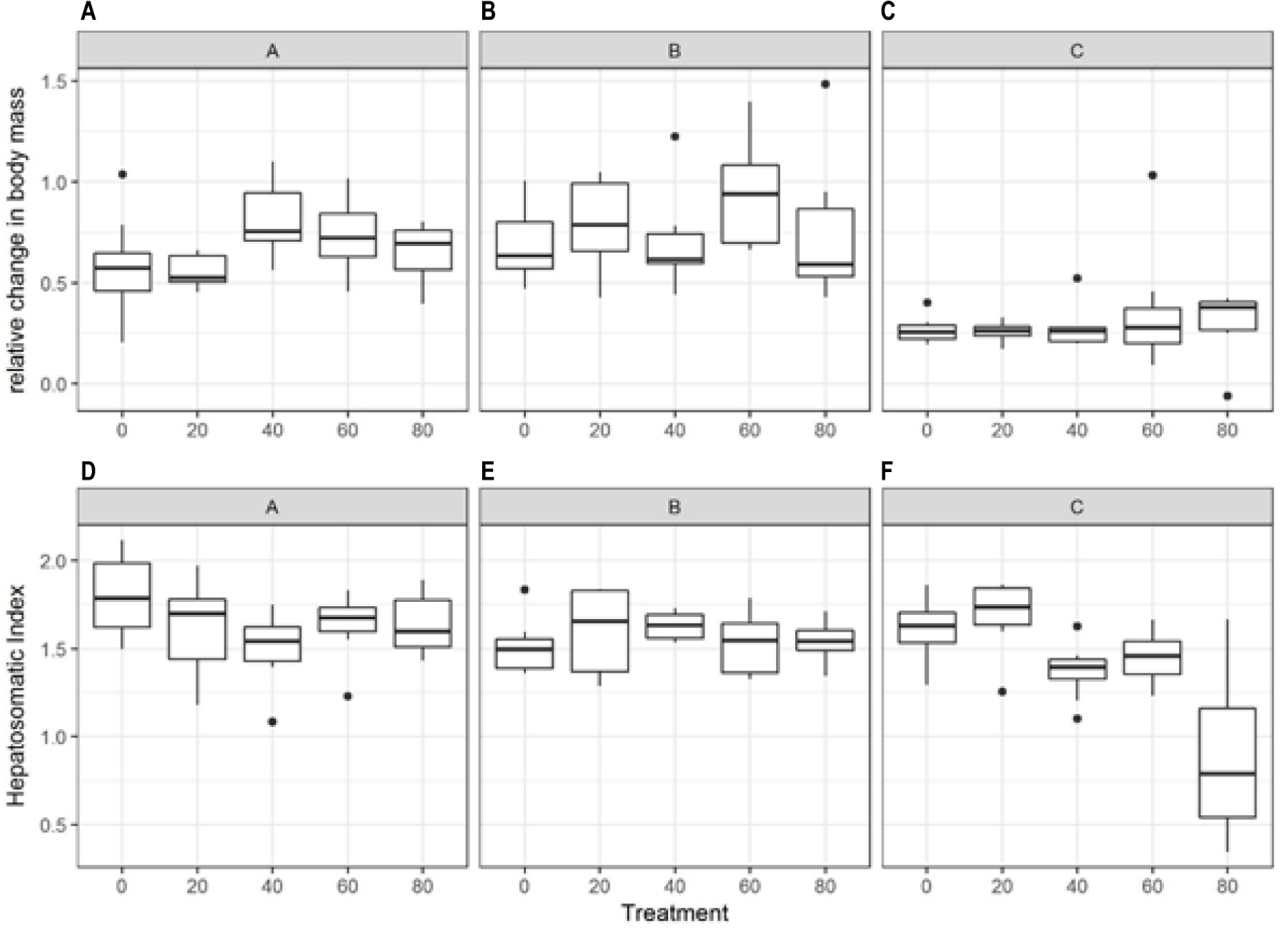
Fish growth and body condition (measured as HSI) during the chronic exposure phase of the experiment. The top panels (A-C) show the change in body mass relative to the start of the chronic exposure, for each clutch. The lower panels (D-F) show the body condition of the fish in the form of the hepatosomatic Index in each treatment and clutch. The mid-line of the boxes represent means with the boxes showing the 25th and 75th percentile and the vertical lines representing the range.

Fish growth during the acute exposure phase of the experiment showed the lowest growth in the higher plastic treatments (Fig 4); these fish were receiving much less food than the control fish. However, the different clutches also reacted significantly differently in this experiment (Table 1). Clutch A and C had negative growth in the highest plastic treatment while clutch B had reduced, but not negative growth. Conversely, the chronic exposure phase showed very little effect of plastic presence (Fig 5). During this phase, the fish that had lost mass during the acute phase seemed to catch up with the rest of their cohort and by the end of the chronic exposure phase (6 weeks) there was no significant difference between treatments in the relative change in body mass. There was a significant difference in the way clutch C responded compared to the other clutches (Table 1), but within clutch there was no significant effect of plastic concentration.

#### Body condition

Based on the difference in length-weight relationship between the start of the chronic exposure and the end of the experiment, there was no significant change in body condition due to the treatments (Table 1). The only exception to this general pattern was observed in clutch A, where there was an increase in body condition in the 40% plastic treatment compared with the control. Based on the hepatosomatic index, there was a general decrease in body condition in higher plastic concentration treatments (Fig 5D–5F). This decrease was most pronounced for clutch C, for which condition was lower in the 80% treatment; however this trend was not consistent across clutches.

### Behaviour

**Fig 6:**
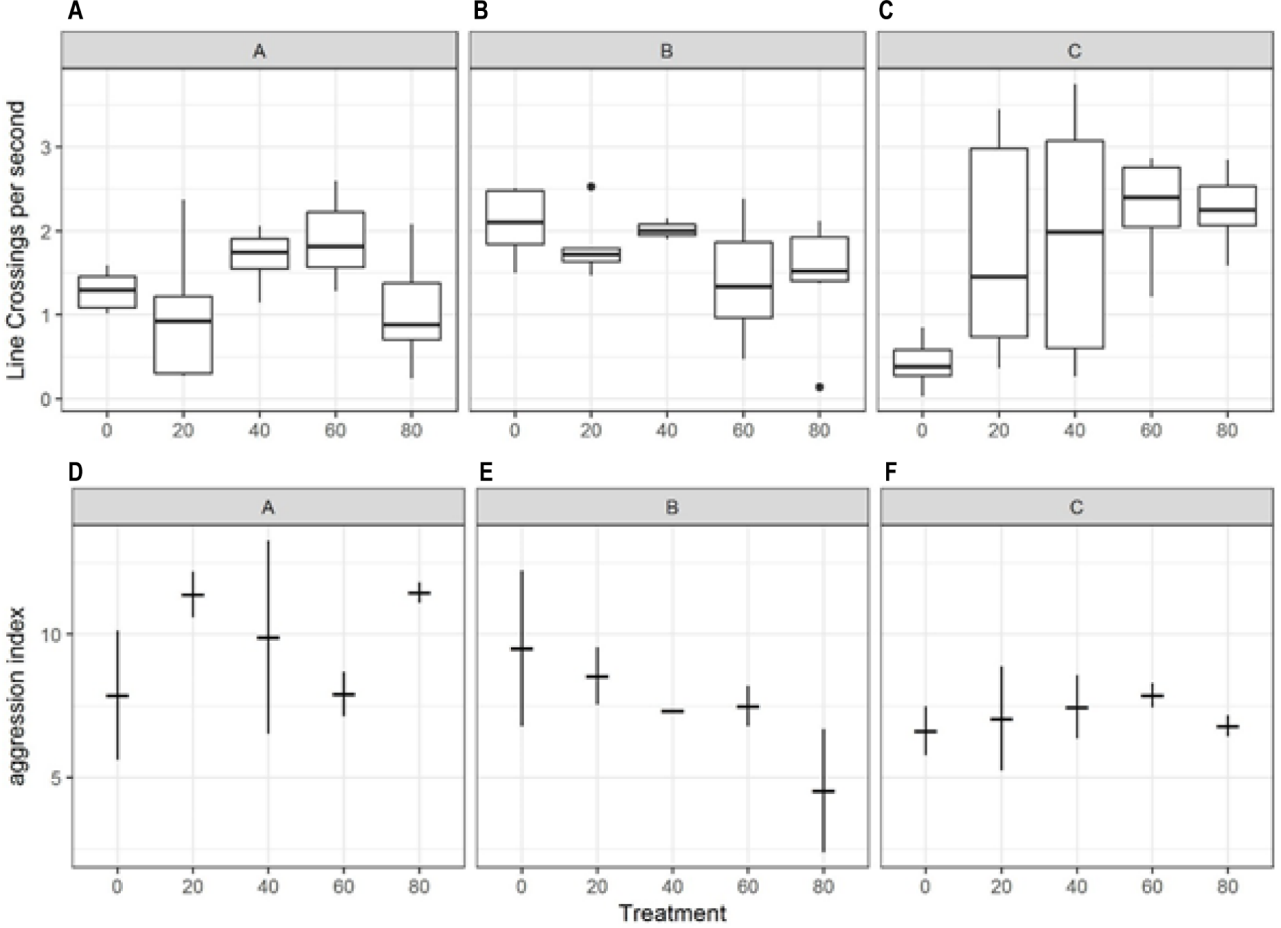
Fish behaviour during exposure to different concentrations of microplastics. The top panels (A-C) show the number of lines crossed per second for each treatment and clutch. The mid-line of the boxes represent means with the boxes showing the 25th and 75th percentile and the vertical lines representing the range. The lower panels (D-F) show the aggression index where the central horizontal line shows the mean and the vertical line indicates the range of values.

Different clutches showed a different behavioural response to the plastic exposure treatments (Fig 6, Table 1). For clutch C, there was a general increase in activity (measured as line crossings) with increasing plastic concentration, but the same trend was not apparent for the other two clutches (Fig 6A). Overall, there was generally high variability in activity among individuals. There was a significant association between the amount of aggression and the number of line crossings observed in each tank (Pearson’s Test, r = 0.8097, df = 7, p = 0.0082). Indeed, the majority of the more aggressive interactions (chases) resulted in a long distance travelled for both the aggressor and the subordinate fish leading to an association between these behavioural metrics. Similar to the observed variation in line crossings, the intensity of aggression was highly variable between tanks within treatments groups. Although aggression was higher, on average, for clutch A (AI of 9.7 compared with 7.5 and 7.2 in clutch B and C, respectively, Fig 6B), ANCOVA with Tukey’s post-hoc test showed that these differences were not statistically significant. There was also some indication for clutch B of a decline in aggression with plastic concentration, but this trend was not statistically significant (Fig 6B).

## Discussion

In this study, we found that when microplastics were the same size as a fish’s normal food, plastic retention in the gut were generally low, and were independent of the concentration of microplastic available in seawater. Moreover, this low level plastic consumption had negligible effects on the growth and body condition of juvenile planktivorous reef fish after 6 weeks of exposure. However, when plastic particles replaced food there was a significant decrease in growth and body condition due to limited food availability. Our results also indicated that plastic consumption and/or plastic presence in seawater did not affect fish foraging behaviour (activity) or aggressive interactions. However, the amount of plastic found in the GI tract was influenced by microplastic particle size, with smaller microplastics much more frequently consumed than larger microplastics, regardless of fish size. These results support the hypothesis that reduced plastics size will increase the consumption of plastics as the ability to distinguish between food and non-food particles decreases.

The low plastic particle retention in the GI tract (up to eight particles per fish) for microplastics 1–2 mm diameter observed in this study suggests that juvenile *A*. *polyacanthus* can recognise and avoid consuming microplastic particles of certain sizes. Although data quantifying microplastic concentrations in coral reef waters are sparse and highly variable among locations, the highest concentrations of microplastics used in this study are generally higher than the concentrations of microplastics found in nature [61]. However, we found no evidence that microplastics presence in the gut was dose-dependent which demonstrates that ingestion of microplastics can occur even at the low concentrations found in the environment. The number of particles in the GI tract of the fish found here is generally consistent with field studies. For example, Lusher et al. [62] found small quantities of microplastic particles (one to 15 per fish) were commonly ingested by fish in the natural environment regardless of fish species and feeding habitat. A similar range of values was found by Neves et al. [10] and Foekema et al. [38], who found only one to four particles per fish, with most fish containing only 1 particle.

Field-sampled fish tended to have mostly plastic fibres in their GI tract, as opposed to particles as assessed in this study, suggesting that fish can more readily recognise and avoid certain types and/or shapes of microplastics. Hermsen et al. [63] suggest many of these fibres could be a result of contamination from sample preparation methods, and this should be considered in future studies. Nevertheless, in our study the particulate microplastics used in the experiments were easily distinguishable from fibres and we found that when plastic particles were less than one quarter the size of normal food (300–125 μm) retention in the GI tract was vastly higher. Collectively, these results indicate that when fish are exposed to a variety of sizes of plastics, smaller plastics will be ingested more readily than larger ones. This dramatic variation in the number of plastics found in the GI tract of different size classes suggests that as plastics get smaller they may be less readily differentiated from normal prey [1, 38, 43]. While we did not quantify the effects of ingestion of these small microplastics, it is likely that very small plastics cause less damage and/or blockage to the digestive system. There is a possibility that fish could reach satiation based on volume not number of particles, therefore, consumption of a many very small particles could have a less impact to the individual, than fewer larger ones. However, the increased surface area to volume ratio of smaller microplastics increases the possibility that associated contaminants or toxins could leach from the microplastics and be absorbed by the fish. Further studies are required to assess whether microplastics will have a larger effect on fish populations as they break up into smaller pieces, as they are more readily consumed and have higher potential to transfer contaminants [64].

Under acute exposure conditions, where microplastic particles replaced food particles, the fish grew slower and lost body condition, and these effects were larger at higher microplastic concentrations. The effects of a reduction in food availability on fish growth and survival are well understood [65]. Indeed, the minimum food requirements for growth of various fish species have been quantified in aquaculture and natural settings [66–68]. Consequently, changes in fish growth and condition during this phase of the experiment likely reflect the reduction in food ration rather than any toxic effects of microplastic exposure. Although we did not measure plastic retention in the gut at the end of the acute phase, plastic retention was low at the chronic phase of the experiment. These results indicate that plastic ingestion is low even when food availability is restricted, and/or that small PET fragments plastics pass through the digestive tract and are evacuated with minimal harm to fish. Our results also show that fish with decreased, and even negative, growth under low food (high microplastic) treatments during the acute phase were the same size, on average, as fish in the high food (low microplastic) treatments by the end of the chronic phase. Such, ‘compensatory’ growth is commonly observed in fish [69]. In our study, fish ‘caught up’ in size within 6 weeks, highlighting that sub-adult fish of this species favour growth at the early stage of life to reduce predation risk for the individual [70]. The literature documents that plankton and plastics are both patchy in nature [71]. Consequently, fish might face intermittent periods of low food rations that have negative effects on fitness by slowing growth rates, and by flow-on effects associated with compensatory growth of juvenile and sub-adult individuals.

### Behaviour

The behavioural measures used in this study (activity and aggression) were directly correlated, suggesting that in aquaria that had more active fish, the fish were being active because of the aggressive interactions taking place. The dominant and most aggressive fish often utilised the shelter, and would chase subordinate fish away from the entrance (KC personal observation). Low [72] reports subordinate fish being driven a total of five metres in a five minute period of field observations of reef fish (Family Pomacentridae). Dominance and space use are tightly correlated, including feeding position [73] and shelter use [74]. The feeding dynamics of a group can also change with changing dominance hierarchy [75, 76]. We found no significant relationship between activity and treatment, with large amounts of variation between and within clutches. There was a slight suggestion that fish are more active at medium levels of exposure. This would support findings of increased aggression at medium feed levels by Toobaie and Grant [77]. It would have been valuable to our study to have behavioural observations before and after exposure, as this would have allowed direct observation of changes to behaviour. We observed vastly different feeding behaviour between individuals and aquaria, suggesting some individuals or groups within a population may be more affected by plastic consumption than others. Indeed, Sparks et al. [78] suggest that a dominance hierarchy can greatly affect the outcome of ecological experiments.

The rate of interactions between individuals and plastics may influence the rate of learning of the individual. There is strong evidence that individual fish learn quickly to avoid anglers in catch and release fisheries, which have strong learning experiences [79]. However, when the plastic particle size is small, we suggest the fish do not have the same learning experience, as the plastics have the potential to simply pass through their GI tract with little to no discomfort. This allows plastics to enter the GI tract, potentially transferring plastics-associated toxins, possibly causing harm to the fish, without an avoidance behaviour to stop it. In most parts of the oceans, fish would currently encounter plastics very rarely, not allowing the learning experience (e.g. < 1 MP m^-3^ in East China Sea [80]). In areas with high microplastics load, for example the North-eastern Pacific (~280 MP m^-3^ [81]), the fish may actually be more equipped to avoid them. The size and shape of particles that could be avoided are probably based on fish species, feeding type, gape, etc. that affect the learning experience of the individual fish.

### Conclusions

We found that juvenile *A*. *polyacanthus* can be tolerant of plastic exposure, finding no significant effect on growth, body condition or behaviour while the plastic particles were the same size as their natural food particles. However, plastic retention vastly increased when the size of the plastic particles was reduced from ~2 mm to 300–125 μm. This is of concern because plastics in the environment fragment into smaller and smaller pieces and, therefore, could become more readily ingested by planktivorous fish.

## Supporting information

S1 File Feeding procedure for the experiments. (DOCX)

S2 File Details of the length-weight relationships of the three clutches. (DOCX)
