# Correction: Effects of microplastic exposure on the body condition and behaviour of planktivorous reef fish (*Acanthochromis polyacanthus*)

**DOI:** 10.1371/journal.pone.0306682

**Published:** 2024-07-02

**Authors:** Kay Critchell, Mia O. Hoogenboom

In Fig 1, the density values are incorrect. Please see the correct Fig 1 here.

**Fig 1:**
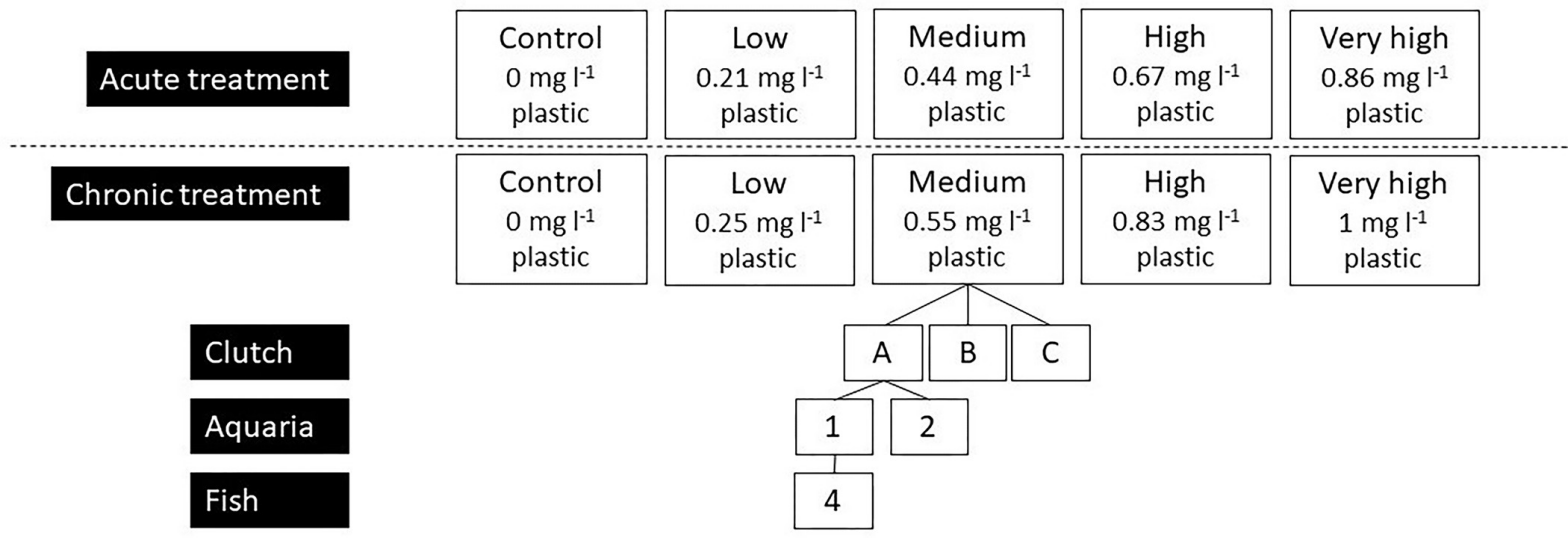
Experimental design for acute and chronic plastic exposure experiment.

Concentrations shown are the mean concentrations for each treatment, as treatment dosage was dependent on tank biomass.

In Overview of approach subsection of Methods, there is an error in the fourth sentence of the third paragraph. The correct sentence is: The fish from each clutch were randomly split into one of five treatments with different microplastic concentrations: control (0 mg l-1 plastic), low (average 0.25 mg l-1 plastic), medium (average 0.55 mg l-1 plastic), high (average 0.83 mg l-1 plastic) and very high (average 1 mg l-1 plastic).

In Acute exposure experiment–food replaced by plastic subsection of Methods, there is an error in the third sentence. The correct sentence is: During this week, the fish (N = 112) received a total ‘food’ allowance between 0.022 g to 0.065 g (0.55–1.6 mg l-1) per feed, depending on aquaria fish biomass, which included different proportions of food and PET (see Table A in S1 File).

In Chronic exposure experiment–plastic dose added to normal food subsection of Methods, there is an error in the second sentence. The correct sentence is: No tank received more than 0.065g (1.6 mg l-1) food per feeding bout, as excess food in the system can reduce water quality.

In Ontogenetic changes in sizes of microplastics ingested subsection of Methods, there is an error in the third sentence. The correct sentence is: The fish were fed twice daily with a diet of commercial pellets (amount calculated based on fish biomass in each aquaria as above) with additional plastic 80% of the food mass (0.5 to 1.3 mg l-1 per feed, see Table B in S1 File).

There is an error in the S1 File. The density values are incorrect. Please see the correct S1 File here.

## Supporting information

S1 File (DOCX)

## References

[1] Critchell K, Hoogenboom MO (2018) Effects of microplastic exposure on the body condition and behaviour of planktivorous reef fish (*Acanthochromis polyacanthus*). PLoS ONE 13(3): e0193308. 10.1371/journal.pone.0193308 29494635 PMC5832226

