# Supplementary material for: Effects of microplastic exposure on the body condition and behaviour of planktivorous reef fish (Acanthochromis polyacanthus)
Source: PLoS One. 2018 Mar 1;13(3):e0193308. doi: 10.1371/journal.pone.0193308 (PMC5832226; doi:10.1371/journal.pone.0193308)
Supplement: S1 File — (DOCX) [file pone.0193308.s001.docx]

S1 Table A: feeding regime for the chronic and acute exposure experiments

| clutch ID | Tank ID | % Diet as plastic | Acute exposure | | week 1 | | week 2 | | week 3 | | week 4 | | week 5 | | week 6 | |
| --- | --- | --- | --- | --- | --- | --- | --- | --- | --- | --- | --- | --- | --- | --- | --- | --- |
|  |  |  | food (mg -l) | plastics (mg l-1) | food (mg -l) | plastics (mg l-1) | food (mg -l) | plastics (mg l-1) | food (mg -l) | plastics (mg l-1) | food (mg -l) | plastics (mg l-1) | food (mg -l) | plastics (mg l-1) | food (mg -l) | plastics (mg l-1) |
| A | 15A | 20 | 0.083 | 0.021 | 0.100 | 0.020 | 0.113 | 0.023 | 0.140 | 0.028 | 0.157 | 0.031 | 0.157 | 0.031 | 0.174 | 0.035 |
| A | 2A | 20 | 0.080 | 0.020 | 0.100 | 0.020 | 0.113 | 0.023 | 0.138 | 0.028 | 0.146 | 0.029 | 0.146 | 0.029 | 0.162 | 0.032 |
| A | 14A | 40 | 0.066 | 0.044 | 0.100 | 0.040 | 0.125 | 0.050 | 0.166 | 0.067 | 0.181 | 0.072 | 0.181 | 0.072 | 0.210 | 0.084 |
| A | 3A | 40 | 0.069 | 0.046 | 0.100 | 0.040 | 0.128 | 0.050 | 0.165 | 0.066 | 0.163 | 0.065 | 0.163 | 0.065 | 0.225 | 0.090 |
| A | 13A | 60 | 0.040 | 0.060 | 0.100 | 0.060 | 0.105 | 0.063 | 0.135 | 0.081 | 0.147 | 0.088 | 0.147 | 0.088 | 0.167 | 0.100 |
| A | 4A | 60 | 0.041 | 0.062 | 0.100 | 0.060 | 0.110 | 0.065 | 0.141 | 0.085 | 0.151 | 0.091 | 0.151 | 0.091 | 0.173 | 0.104 |
| A | 12A | 80 | 0.022 | 0.086 | 0.100 | 0.080 | 0.110 | 0.088 | 0.140 | 0.112 | 0.145 | 0.116 | 0.145 | 0.116 | 0.168 | 0.134 |
| A | 5A | 80 | 0.018 | 0.071 | 0.100 | 0.080 | 0.090 | 0.070 | 0.116 | 0.092 | 0.123 | 0.098 | 0.123 | 0.098 | 0.140 | 0.112 |
| A | 11A | Control | 0.104 | 0.000 | 0.100 | 0.000 | 0.113 | 0.000 | 0.130 | 0.000 | 0.134 | 0.000 | 0.134 | 0.000 | 0.152 | 0.000 |
| A | 1A | Control | 0.098 | 0.000 | 0.100 | 0.000 | 0.108 | 0.000 | 0.132 | 0.000 | 0.146 | 0.000 | 0.146 | 0.000 | 0.171 | 0.000 |
| B | 19B | 20 | 0.045 | 0.011 | 0.100 | 0.020 | 0.065 | 0.013 | 0.082 | 0.016 | 0.089 | 0.018 | 0.089 | 0.018 | 0.106 | 0.021 |
| B | 7B | 20 | 0.059 | 0.015 | 0.100 | 0.020 | 0.080 | 0.015 | 0.100 | 0.020 | 0.106 | 0.021 | 0.106 | 0.021 | 0.125 | 0.025 |
| B | 18B | 40 | 0.041 | 0.027 | 0.100 | 0.040 | 0.078 | 0.030 | 0.096 | 0.039 | 0.103 | 0.041 | 0.103 | 0.041 | 0.124 | 0.050 |
| B | 8B | 40 | 0.035 | 0.023 | 0.100 | 0.040 | 0.063 | 0.025 | 0.070 | 0.028 | 0.076 | 0.030 | 0.076 | 0.030 | 0.093 | 0.037 |
| B | 17B | 60 | 0.035 | 0.053 | 0.100 | 0.060 | 0.093 | 0.055 | 0.122 | 0.073 | 0.138 | 0.083 | 0.138 | 0.083 | 0.175 | 0.105 |
| B | 9B | 60 | 0.030 | 0.045 | 0.100 | 0.060 | 0.083 | 0.048 | 0.099 | 0.059 | 0.106 | 0.063 | 0.106 | 0.063 | 0.127 | 0.076 |
| B | 10B | 80 | 0.015 | 0.058 | 0.100 | 0.080 | 0.075 | 0.058 | 0.090 | 0.072 | 0.093 | 0.074 | 0.093 | 0.074 | 0.108 | 0.087 |
| B | 16B | 80 | 0.013 | 0.052 | 0.100 | 0.080 | 0.068 | 0.055 | 0.086 | 0.069 | 0.088 | 0.071 | 0.088 | 0.071 | 0.107 | 0.085 |
| B | 20B | Control | 0.091 | 0.000 | 0.100 | 0.000 | 0.110 | 0.000 | 0.137 | 0.000 | 0.149 | 0.000 | 0.149 | 0.000 | 0.185 | 0.000 |
| B | 6B | Control | 0.062 | 0.000 | 0.100 | 0.000 | 0.070 | 0.000 | 0.087 | 0.000 | 0.094 | 0.000 | 0.094 | 0.000 | 0.110 | 0.000 |
| C | 2C | 20 | 0.125 | 0.030 | 0.150 | 0.030 | 0.157 | 0.031 | 0.163 | 0.033 | 0.163 | 0.033 | 0.163 | 0.033 | 0.163 | 0.033 |
| C | 7C | 20 | 0.118 | 0.028 | 0.138 | 0.028 | 0.145 | 0.029 | 0.154 | 0.031 | 0.158 | 0.032 | 0.163 | 0.033 | 0.163 | 0.033 |
| C | 3C | 40 | 0.098 | 0.065 | 0.163 | 0.065 | 0.163 | 0.065 | 0.163 | 0.065 | 0.163 | 0.065 | 0.163 | 0.065 | 0.163 | 0.065 |
| C | 8C | 40 | 0.090 | 0.059 | 0.148 | 0.058 | 0.148 | 0.059 | 0.158 | 0.063 | 0.164 | 0.065 | 0.163 | 0.065 | 0.163 | 0.065 |
| C | 4C | 60 | 0.060 | 0.093 | 0.150 | 0.090 | 0.152 | 0.091 | 0.162 | 0.097 | 0.163 | 0.098 | 0.163 | 0.098 | 0.163 | 0.098 |
| C | 9C | 60 | 0.060 | 0.090 | 0.143 | 0.085 | 0.146 | 0.088 | 0.154 | 0.093 | 0.162 | 0.097 | 0.163 | 0.098 | 0.163 | 0.098 |
| C | 10C | 80 | 0.033 | 0.130 | 0.150 | 0.120 | 0.162 | 0.129 | 0.163 | 0.130 | 0.163 | 0.130 | 0.163 | 0.130 | 0.163 | 0.130 |
| C | 5C | 80 | 0.030 | 0.118 | 0.135 | 0.108 | 0.144 | 0.115 | 0.154 | 0.123 | 0.163 | 0.130 | 0.163 | 0.130 | 0.163 | 0.130 |
| C | 1C | Control | 0.145 | 0.000 | 0.140 | 0.000 | 0.145 | 0.000 | 0.149 | 0.000 | 0.156 | 0.000 | 0.162 | 0.000 | 0.163 | 0.000 |
| C | 6C | Control | 0.140 | 0.000 | 0.135 | 0.000 | 0.142 | 0.000 | 0.148 | 0.000 | 0.153 | 0.000 | 0.162 | 0.000 | 0.163 | 0.000 |

S1 Table B: feeding regime for the particle size experiment

| Tank Number | Fish Size Class | Plastic size class | Food (mg l^-1^) | plastics (mg l^-1^) |
| --- | --- | --- | --- | --- |
| 3 | Small | Small | 0.065 | 0.05 |
| 4 | Large | Large | 0.14 | 0.1 |
| 5 | Small | Small | 0.0775 | 0.06 |
| 6 | Large | Large | 0.1625 | 0.13 |
| 7 | Large | Medium | 0.15 | 0.12 |
| 8 | Small | Medium | 0.1 | 0.08 |
| 9 | Small | Small | 0.085 | 0.0675 |
| 10 | Small | Large | 0.1325 | 0.105 |
| 11 | Small | Large | 0.135 | 0.1075 |
| 12 | Large | Large | 0.1625 | 0.13 |
| 13 | Large | Medium | 0.1475 | 0.1175 |
| 14 | Small | Medium | 0.085 | 0.0675 |
| 15 | Large | Small | 0.1625 | 0.13 |
| 16 | Large | Medium | 0.1625 | 0.130 |
| 17 | Large | Small | 0.1625 | 0.130 |
| 18 | Small | Medium | 0.095 | 0.075 |
| 19 | Large | Small | 0.1625 | 0.130 |
| 20 | Small | Large | 0.11 | 0.088 |
