# Supplementary material for: Effects of microplastic exposure on the body condition and behaviour of planktivorous reef fish (Acanthochromis polyacanthus)
Source: PLoS One. 2018 Mar 1;13(3):e0193308. doi: 10.1371/journal.pone.0193308 (PMC5832226; doi:10.1371/journal.pone.0193308)
Supplement: S2 File — (DOCX) [file pone.0193308.s002.docx]

S2 Table A: table of ANOVA results with the response variable initial weight

|  | Df | Sum Sq | Mean Sq | F value | Pr(>F) |
| --- | --- | --- | --- | --- | --- |
| Initial Length | 1 | 20.1960 | 20.1960 | 3632.957 | < 2.2e-16 |
| Clutch | 2 | 0.2187 | 0.1093 | 19.668 | 5.440e-08 |
| Initial Length : Clutch | 2 | 0.3750 | 0.1875 | 33.727 | 4.617e-12 |
| Residuals | 106 | 0.5893 | 0.0056 |  |  |


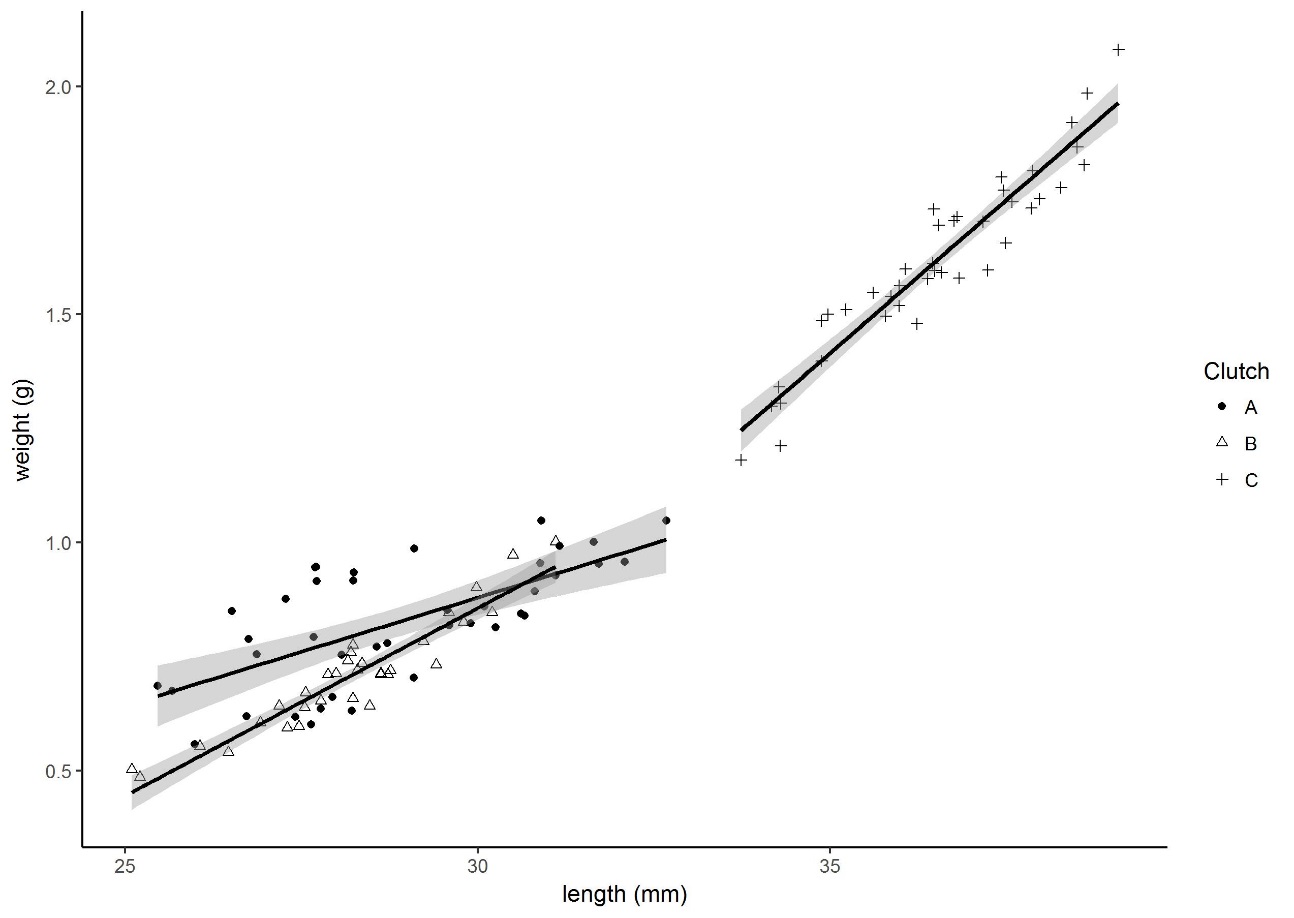


S2 Figure A: length weight relationships of the 3 clutches at the start of the experiment.

S2 Table B: coefficients of the three clutches

| Clutch | Intercept | slope |
| --- | --- | --- |
| A | -0.54460085 | 0.04746250 |
| B | -1.06841405 | 0.03484301 |
| C | -2.73881573 | 0.08678282 |
