# Supplementary material for: Correction: Effects of microplastic exposure on the body condition and behaviour of planktivorous reef fish (Acanthochromis polyacanthus)
Source: PLoS One. 2024 Jul 2;19(7):e0306682. doi: 10.1371/journal.pone.0306682 (PMC11218960; doi:10.1371/journal.pone.0306682)
Supplement: S1 File — (DOCX) [file pone.0306682.s001.docx]

S1 Table A: feeding regime for the chronic and acute exposure experiments

| clutch ID | Tank ID | % Diet as plastic | Acute exposure | | week 1 | | week 2 | | week 3 | | week 4 | | week 5 | | week 6 | |
| --- | --- | --- | --- | --- | --- | --- | --- | --- | --- | --- | --- | --- | --- | --- | --- | --- |
|  |  |  | food (mg -l) | plastics (mg l-1) | food (mg -l) | plastics (mg l-1) | food (mg -l) | plastics (mg l-1) | food (mg -l) | plastics (mg l-1) | food (mg -l) | plastics (mg l-1) | food (mg -l) | plastics (mg l-1) | food (mg -l) | plastics (mg l-1) |
| A | 15A | 20 | 0.83 | 0.21 | 1.00 | 0.20 | 1.13 | 0.23 | 1.40 | 0.28 | 1.57 | 0.31 | 1.57 | 0.31 | 1.74 | 0.35 |
| A | 2A | 20 | 0.80 | 0.20 | 1.00 | 0.20 | 1.13 | 0.23 | 1.38 | 0.28 | 1.46 | 0.29 | 1.46 | 0.29 | 1.62 | 0.32 |
| A | 14A | 40 | 0.66 | 0.44 | 1.00 | 0.40 | 1.25 | 0.50 | 1.66 | 0.67 | 1.81 | 0.72 | 1.81 | 0.72 | 2.10 | 0.84 |
| A | 3A | 40 | 0.69 | 0.46 | 1.00 | 0.40 | 1.28 | 0.50 | 1.65 | 0.66 | 1.63 | 0.65 | 1.63 | 0.65 | 2.25 | 0.90 |
| A | 13A | 60 | 0.40 | 0.60 | 1.00 | 0.60 | 1.05 | 0.63 | 1.35 | 0.81 | 1.47 | 0.88 | 1.47 | 0.88 | 1.67 | 1.00 |
| A | 4A | 60 | 0.41 | 0.62 | 1.00 | 0.60 | 1.10 | 0.65 | 1.41 | 0.85 | 1.51 | 0.91 | 1.51 | 0.91 | 1.73 | 1.04 |
| A | 12A | 80 | 0.22 | 0.86 | 1.00 | 0.80 | 1.10 | 0.88 | 1.40 | 1.12 | 1.45 | 1.16 | 1.45 | 1.16 | 1.68 | 1.34 |
| A | 5A | 80 | 0.18 | 0.71 | 1.00 | 0.80 | 0.90 | 0.70 | 1.16 | 0.92 | 1.23 | 0.98 | 1.23 | 0.98 | 1.40 | 1.12 |
| A | 11A | Control | 1.04 | 0.00 | 1.00 | 0.00 | 1.13 | 0.00 | 1.30 | 0.00 | 1.34 | 0.00 | 1.34 | 0.00 | 1.52 | 0.00 |
| A | 1A | Control | 0.98 | 0.00 | 1.00 | 0.00 | 1.08 | 0.00 | 1.32 | 0.00 | 1.46 | 0.00 | 1.46 | 0.00 | 1.71 | 0.00 |
| B | 19B | 20 | 0.45 | 0.11 | 1.00 | 0.20 | 0.65 | 0.13 | 0.82 | 0.16 | 0.89 | 0.18 | 0.89 | 0.18 | 1.06 | 0.21 |
| B | 7B | 20 | 0.59 | 0.15 | 1.00 | 0.20 | 0.80 | 0.15 | 1.00 | 0.20 | 1.06 | 0.21 | 1.06 | 0.21 | 1.25 | 0.25 |
| B | 18B | 40 | 0.41 | 0.27 | 1.00 | 0.40 | 0.78 | 0.30 | 0.96 | 0.39 | 1.03 | 0.41 | 1.03 | 0.41 | 1.24 | 0.50 |
| B | 8B | 40 | 0.35 | 0.23 | 1.00 | 0.40 | 0.63 | 0.25 | 0.70 | 0.28 | 0.76 | 0.30 | 0.76 | 0.30 | 0.93 | 0.37 |
| B | 17B | 60 | 0.35 | 0.53 | 1.00 | 0.60 | 0.93 | 0.55 | 1.22 | 0.73 | 1.38 | 0.83 | 1.38 | 0.83 | 1.75 | 1.05 |
| B | 9B | 60 | 0.30 | 0.45 | 1.00 | 0.60 | 0.83 | 0.48 | 0.99 | 0.59 | 1.06 | 0.63 | 1.06 | 0.63 | 1.27 | 0.76 |
| B | 10B | 80 | 0.15 | 0.58 | 1.00 | 0.80 | 0.75 | 0.58 | 0.90 | 0.72 | 0.93 | 0.74 | 0.93 | 0.74 | 1.08 | 0.87 |
| B | 16B | 80 | 0.13 | 0.52 | 1.00 | 0.80 | 0.68 | 0.55 | 0.86 | 0.69 | 0.88 | 0.71 | 0.88 | 0.71 | 1.07 | 0.85 |
| B | 20B | Control | 0.91 | 0.00 | 1.00 | 0.00 | 1.10 | 0.00 | 1.37 | 0.00 | 1.49 | 0.00 | 1.49 | 0.00 | 1.85 | 0.00 |
| B | 6B | Control | 0.62 | 0.00 | 1.00 | 0.00 | 0.70 | 0.00 | 0.87 | 0.00 | 0.94 | 0.00 | 0.94 | 0.00 | 1.10 | 0.00 |
| C | 2C | 20 | 1.25 | 0.30 | 1.50 | 0.30 | 1.57 | 0.31 | 1.63 | 0.33 | 1.63 | 0.33 | 1.63 | 0.33 | 1.63 | 0.33 |
| C | 7C | 20 | 1.18 | 0.28 | 1.38 | 0.28 | 1.45 | 0.29 | 1.54 | 0.31 | 1.58 | 0.32 | 1.63 | 0.33 | 1.63 | 0.33 |
| C | 3C | 40 | 0.98 | 0.65 | 1.63 | 0.65 | 1.63 | 0.65 | 1.63 | 0.65 | 1.63 | 0.65 | 1.63 | 0.65 | 1.63 | 0.65 |
| C | 8C | 40 | 0.90 | 0.59 | 1.48 | 0.58 | 1.48 | 0.59 | 1.58 | 0.63 | 1.64 | 0.65 | 1.63 | 0.65 | 1.63 | 0.65 |
| C | 4C | 60 | 0.60 | 0.93 | 1.50 | 0.90 | 1.52 | 0.91 | 1.62 | 0.97 | 1.63 | 0.98 | 1.63 | 0.98 | 1.63 | 0.98 |
| C | 9C | 60 | 0.60 | 0.90 | 1.43 | 0.85 | 1.46 | 0.88 | 1.54 | 0.93 | 1.62 | 0.97 | 1.63 | 0.98 | 1.63 | 0.98 |
| C | 10C | 80 | 0.33 | 1.30 | 1.50 | 1.20 | 1.62 | 1.29 | 1.63 | 1.30 | 1.63 | 1.30 | 1.63 | 1.30 | 1.63 | 1.30 |
| C | 5C | 80 | 0.30 | 1.18 | 1.35 | 1.08 | 1.44 | 1.15 | 1.54 | 1.23 | 1.63 | 1.30 | 1.63 | 1.30 | 1.63 | 1.30 |
| C | 1C | Control | 1.45 | 0.00 | 1.40 | 0.00 | 1.45 | 0.00 | 1.49 | 0.00 | 1.56 | 0.00 | 1.62 | 0.00 | 1.63 | 0.00 |
| C | 6C | Control | 1.40 | 0.00 | 1.35 | 0.00 | 1.42 | 0.00 | 1.48 | 0.00 | 1.53 | 0.00 | 1.62 | 0.00 | 1.63 | 0.00 |

S1 Table B: feeding regime for the particle size experiment

| Tank Number | Fish Size Class | Plastic size class | Food (mg l-1) | plastics (mg l-1) |
| --- | --- | --- | --- | --- |
| 3 | Small | Small | 0.65 | 0.50 |
| 4 | Large | Large | 1.40 | 1.00 |
| 5 | Small | Small | 0.78 | 0.60 |
| 6 | Large | Large | 1.63 | 1.30 |
| 7 | Large | Medium | 1.50 | 1.20 |
| 8 | Small | Medium | 1.00 | 0.80 |
| 9 | Small | Small | 0.85 | 0.68 |
| 10 | Small | Large | 1.33 | 1.05 |
| 11 | Small | Large | 1.35 | 1.08 |
| 12 | Large | Large | 1.63 | 1.30 |
| 13 | Large | Medium | 1.48 | 1.18 |
| 14 | Small | Medium | 0.85 | 0.68 |
| 15 | Large | Small | 1.63 | 1.30 |
| 16 | Large | Medium | 1.63 | 1.30 |
| 17 | Large | Small | 1.63 | 1.30 |
| 18 | Small | Medium | 0.95 | 0.75 |
| 19 | Large | Small | 1.63 | 1.30 |
| 20 | Small | Large | 1.10 | 0.88 |
